# Importance of Tissue Selection for Genetic Testing: Detection of a Terminal 18q Deletion after Stem Cell Transplantation

**DOI:** 10.1002/mdc3.12931

**Published:** 2020-04-03

**Authors:** Tina Mainka, Saskia Biskup, Andrea A. Kühn, Christine Klein, Katja Lohmann, Christos Ganos

**Affiliations:** ^1^ Department of Neurology Charité University Medicine Berlin Berlin Germany; ^2^ Berlin Institute of Health Berlin Germany; ^3^ Center for Genomics and Transcriptomics Tübingen Germany; ^4^ Institute of Neurogenetics University of Lübeck Lübeck Germany

**Keywords:** 18q deletion, skin photosensitivity, stem cell transplantation, genetic testing, cerebellar ataxia

https://onlinelibrary.wiley.com/page/journal/23301619/homepage/mdc312931-sup-v001.htm

A variety of genetic alterations can lead to complex neurological syndromes.^1^ Despite being clinically and genetically heterogeneous, in some cases, characteristic clinical features can guide diagnostic reasoning to specific genetic mutations. However, clinical signs often overlap, and it can be notoriously difficult to reach a conclusive diagnosis based on clinical investigation alone. Despite the advent of new genetic techniques, such as multigene panels and whole‐exome sequencing, that has increased the diagnostic yield, the genetic underpinnings of many complex cases remain elusive. The absence of a relevant family history, phenotypic heterogeneity, and the selection of inappropriate investigative tools (eg, wrong gene panel) constitute some of the factors contributing to diagnostic failure.

Here we highlight the importance of tissue selection as an additional and perhaps underrecognized factor to inform genetic testing and reach diagnosis in the case of a patient with a complex neurological phenotype who has also undergone allogeneic stem cell transplantation (SCT) due to acute lymphoblastic leukemia (ALL). Only genetic investigations from the patient's saliva, but not blood (stem cell donor's DNA) led to the correct genetic diagnosis of a terminal 18q deletion.

## Case Report

A 50‐year‐old male presented with progressive difficulties in precise hand movements, loss of balance, and speech impairment. There was no family history, and delivery was uneventful. During childhood, he showed delayed developmental milestones and below‐average school performance. Problems with balance and falls were noted. During adolescence, he was diagnosed with epilepsy and treated with carbamazepine for several years. He has been seizure‐free for 3 decades without medication. At the age of 44 years, the patient received allogeneic SCT and prophylactic cranial radiotherapy for ALL.

**Figure 1:**
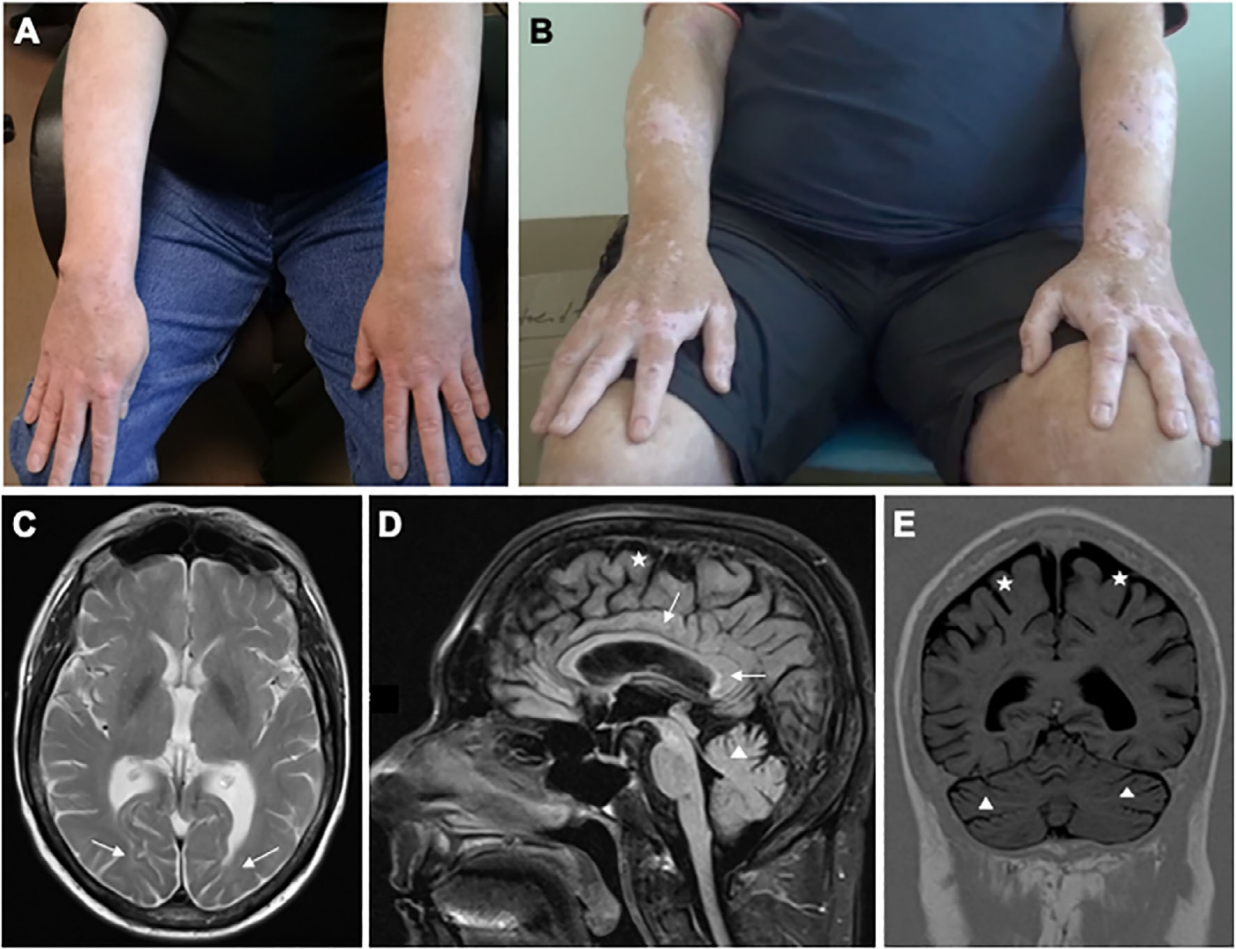
Upper panel: (A) circumscribed, depigmented maculae and patches on the upper extremity. (B) Skin with remarkable hyperpigmentation after short‐time sun exposure. The circumscribed depigmented areas are more pronounced, and some of them show erythema. Lower panel: magnetic resonance imaging at the time of presentation. Mild leukoencephalopathy is noted (C, T2‐weighted axial image, D, fluid attenuated inversion recovery sagittal image, see arrows). Mild generalized brain atrophy is seen (D, E, indicated for the cerebellum [△] and supratentorial brain structures [**★**]).

On examination (see Video S1), facial dysmorphism with prominent nasal bridge, long and flat philtrum, and pointy ears with tightly attached earlobes were noted. Oculomotor examination revealed saccadic pursuit, saccadic dysmetria, and inability to initiate internally generated vertical saccades (oculomotor apraxia for the vertical plane). He had slurred, cerebellar speech. Dystonic posturing of the upper limbs as well as irregular, jerky action tremor, and intermittent myoclonic jerks were noted. Finger–nose tests and heel–knee–shin tests were ataxic. There was no neuropathy. Vitiligo was noted (Fig. 1A). Magnetic resonance imaging revealed mild brain atrophy and mild supratentorial leukoencephalopathy (Fig. 1C–E). Due to the syndromic presentation, array‐based comparative genomic hybridization was undertaken. Given the prior history of allogeneic SCT, both blood and buccal swabs were sent for genetic testing. A ∼4Mb deletion in the terminal long arm of chromosome 18 (18q23; deletion includes markers in chr18:74,285,842_78,010,032) was found. The deletion was only present in the buccal swab, but not in the patient's blood sample. Segregation analysis of the 2 remaining and unaffected relatives (mother and sister) showed no deletion. Exome sequencing from the buccal sample was also performed to exclude mutations in other known genes associated with complex cerebellar ataxia or oculomotor abnormalities and was unrevealing.

## Discussion

The phenotypic spectrum of terminal 18q deletions is broad and encompasses neurodevelopmental disorders as well as ataxia, dystonia, seizures, facial dysmorphism, and strabismus.^2^ Skin conditions, such as ichthyosis and vitiligo, have also been reported.^3^ Our case with complex cerebellar ataxia, intermittent myoclonus, and oculomotor apraxia further expands the range of possible clinical presentations of terminal 18q‐deletion syndrome. Although we cannot exclude an additional effect of cranial radiotherapy in aggravating our patient's symptoms, the characteristic history of delayed developmental milestones, seizures, movement disorders, vitiligo, and facial dysmorphism are all in line with previous reports in literature. The deleted region of about 4Mb comprises 16 protein‐coding genes including myelin basic protein (*MBP*) and *CTDP1* (carboxy‐terminal domain phosphatase 1, biallelic mutations cause a syndrome of congenital cataracts with facial dysmorphism and neuropathy, CCFDN).^4^

Of note, the genetic diagnosis could only be reached with the appropriate tissue selection. Indeed, in movement disorders and neurogenetics practice, patients who have received allogeneic SCT are rare, and standard DNA investigations are based on blood samples. It is paramount, therefore, to be aware that in such cases, genetic investigations should not be performed in blood but other tissues, such as buccal swabs or hair follicles, to avoid false negatives.^5^ Although this practice is well established in DNA forensics,^5^ the discrepancy between the normal results stemming from blood samples (allogeneic SCT donor's DNA) and that from that of the buccal swab (patient's DNA) is highlighted in our patient. To conclude, we here report a patient who presented with a complex neurological syndrome due to a terminal 18q deletion. The deletion syndrome could only be identified upon appropriate tissue selection as the patient had previously undergone allogeneic SCT.

## Author Roles

(1) Research Project: A. Clinical Assessment of the Patient, B. Genetic Analysis, C. Interpretation of Genetic Analysis; (2) Manuscript Preparation: A. Collection and Editing of Video Material, B. Writing of the First Draft, C. Review and Critique.

T.M.: 1A, 1C, 2A, 2B

C.G.: 1A, 1C, 2A, 2B

S.B.: 1B, 1C, 2C

C.K.: 1C, 2C

A.K.K.: 1C, 2C

K.L.: 1C, 2C

## Disclosures

**Ethical Compliance Statement:** The study was conducted in accordance with the Declaration of Helsinki. The authors confirm that the approval of an institutional review board was not required for this work. The patient gave informed consent including being videotaped for publication online.

**Funding Sources and Conflict of Interest**: CG received academic research support from the VolkswagenStiftung (Freigeist Fellowship). The authors declare that there are no conflicts of interest relevant to this work.

**Financial Disclosures for the Previous 12 Months:** T.M. receives royalties from Urban Fischer in Elsevier Verlag and honoraria for lectures from GE Healthcare. T.M. is a participant in the Berlin Institute of Health–Charité Clinician Scientist Program funded by the Charité‐Universitätsmedizin Berlin and the Berlin Institute of Health. S.B. reports no disclosures. C.K. serves as a medical advisor to Centogene for genetic testing reports on movement disorders and dementia excluding Parkinson's disease. A.A.K. is a consultant to Boston Scientific and has received honoraria for speaking from Medtronic, Boston Scientific, and Abbott and grants from Medtronic, all outside the submitted work. K.L. receives funding from the German Research Foundation (FOR2488), the Federal Ministry of Education and Sciences, the Movement Disorder Society, and the Damp‐Foundation. C.G. holds research grants from the VolkswagenStiftung (Freigeist Fellowship) and the German Parkinson Society and was also supported by the Deutsche Forschungsgemeinschaft (GA2031/1‐1 and GA2031/1‐2).

## Supporting information


**Video S1.** Clinical examination. The 50‐year‐old patient shows broken smooth pursuit and difficulties to initiate internal vertical saccades. His speech is dysarthric. Dystonic posturing of the upper limbs is noted. There is an irregular, jerky tremor of the upper extremity with intermittent jerks of both arms. The gait is ataxic, and tandem walk is barely possible. The skin has a patchy appearance between areas with hyperpigmentation and hypopigmentation, also showing erythematous changes after sun exposure.
